# Systematic Handling of Environmental Fate Data for Model Development—Illustrated for the Case of Biodegradation Half-Life Data

**DOI:** 10.1021/acs.estlett.3c00526

**Published:** 2023-09-26

**Authors:** Jasmin Hafner, Kathrin Fenner, Andreas Scheidegger

**Affiliations:** † Swiss Federal Institute of Aquatic Science and Technology (Eawag), 8600 Dübendorf, Zürich, Switzerland; ‡ University of Zürich, 8057 Zürich, Switzerland

**Keywords:** environmental fate data, biodegradation half-lives, soil, censored data, uncertainty, Bayesian inference

## Abstract

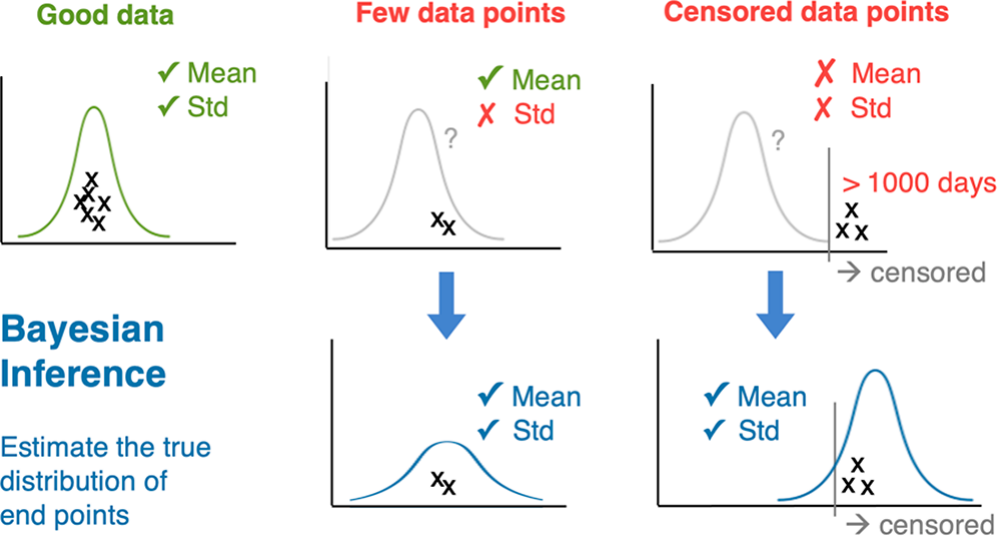

The assessment of environmental hazard indicators such as persistence, mobility, toxicity, or bioaccumulation of chemicals often results in highly variable experimental outcomes. Persistence is particularly affected due to a multitude of influencing environmental factors, with biodegradation experiments resulting in half-lives spanning several orders of magnitude. Also, half-lives may lie beyond the limits of reliable half-life quantification, and the number of available data points per substance may vary considerably, requiring a statistically robust approach for the characterization of data. Here, we apply Bayesian inference to address these challenges and characterize the distributions of reported soil half-lives. Our model estimates the mean, standard deviation, and corresponding uncertainties from a set of reported half-lives experimentally obtained for a single substance. We apply our inference model to 893 pesticides and pesticide transformation products with experimental soil half-lives of varying data quantity and quality, and we infer the half-life distribution for each compound. By estimating average half-lives, their experimental variability, and the uncertainty of the estimations, we provide a reliable data source for building predictive models, which are urgently needed by regulatory authorities to manage existing chemicals and by industry to design benign, nonpersistent chemicals. Our approach can be readily adapted for other environmental hazard indicators.

## Introduction

In the EU, different chemical regulations require different levels of hazard and risk assessment, partially depending on the amounts of substances used and produced.^1−4^ The recently amended regulation for chemicals, labeling, and packaging (CLP) in Europe now requires hazard assessment covering toxicity, bioaccumulation, persistence, and mobility for substances and mixtures introduced into the EU market, to prioritize those with great potential for negative environmental impacts.^5^ Experimental determination of these hazard indicators is costly and time-consuming, and the necessary data for a confident hazard assessment are missing for many substances on the market.^6^ Computational models that predict environmental hazard indicators are necessary to fill data gaps for existing chemicals and to screen for environmentally safe chemicals during the industrial research and development of new chemicals,^7^ ultimately supporting the phase-out of harmful chemicals on the market. However, model training requires reliable and abundant experimental data of sufficient quality, which is scarce, in particular for biodegradation end points.

In addition to the scarcity of data, the main issue is the high variability of environmental hazard indicator data. For example, OECD 307 studies require experimental determination of biotransformation half-lives of a substance in three different types of soil.^8^ While testing in diverse soils is considered necessary to draw general conclusions about the persistence of a chemical in the environment, this will lead to variability in the determined end point data. In the case of soil biodegradation, sources of variability include physicochemical parameters (e.g., temperature and acidity), soil characteristics (e.g., soil texture, cation exchange capacity, biomass content, and organic carbon content), and the composition, activity, and enzymatic potential of the microbial community. Other factors that contribute to the variability of biodegradation data include low but environmentally relevant concentrations of spiked chemicals entailing larger analytical errors and the kinetic model used to derive half-lives from concentration–time series.^9^ While in most cases (pseudo) first-order kinetics are assumed and used to estimate half-lives, some studies employ different kinetic models, typically assuming a fast and a slow degradation phase, to derive degradation half-lives. Hence, the resulting half-life estimates depend on the choice of kinetic models leading to considerable methodological uncertainty. If many experimental values for a single substance are available, then the total variability can be statistically determined as the product of the natural variability of the environmental samples and the methodological uncertainty. However, if only a single or few data points are reported, the total variability remains unknown or uncertain, respectively.

Other issues are experimental outcomes that lie beyond the limits of what can be reliably determined given the specific experimental setup, leading to so-called censored values, i.e., values that can be given as only “smaller than” (left-censored) or “larger than” (right-censored) the reporting limit. A popular strategy is to remove censored values from the data set. However, this introduces statistical bias, and when experimental data are scarce and expensive, removing censored values means further reducing an already small data set.^10^ For persistence assessment, right- or left-censored values indicate that the substance is highly recalcitrant or highly biodegradable, respectively, and thus, its structure is particularly informative for machine learning models that predict biodegradation from molecular structure [i.e., Quantitative Structure Biodegradation Relationships (QSBRs)].

Here, we propose a procedure for handling variable environmental end point data and demonstrate it on soil biotransformation half-life data. We use Bayesian inference to derive the distribution that describes our knowledge about the true half-life distribution, including censored data points. Bayesian inference is a well-established statistical approach for describing data distributions, and it can be applied in cases in which descriptive statistics fail (e.g., low data regimes and censored data).^11,12^ Bayesian inference combines data points and prior assumptions encoded as probability distributions to calculate a posterior distribution, which estimates the true distribution underlying the data in a consistent manner across many different compounds with varying data quality and quantity. We apply our procedure to enhance the quality and reliability of a data set of 6309 experimental soil biotransformation half-lives for 893 pesticides and pesticide transformation products. The data are obtained from the EAWAG-SOIL package on enviPath, which was previously extracted from publicly available regulatory reports.^13^

## Materials and Methods

### Extraction of Data from enviPath

The reported soil biotransformation half-lives were downloaded from enviPath in April 2023 using the workflow available on GitHub (https://github.com/FennerLabs/pepper). The workflow is encoded in Python, and requests to the database are performed via the enviPath-python Application Programming Interface (API) available at https://git.envipath.com/enviPath/enviPath-python.

For each substance in the data set, one or more reported half-life values are available. Each reported half-life resulted from a single experiment and was calculated from a concentration–time series using kinetic models. A substance can have several reported half-lives obtained by different researchers in different years under different experimental conditions and calculated using different kinetic assumptions. For each compound in the EAWAG-SOIL package (https://envipath.org/package/5882df9c-dae1-4d80-a40e-db4724271456), all reported soil biodegradation half-life values were extracted, including available experimental metadata. Compound entries without any associated half-lives were not considered. The following metadata were collected for each reported half-life: study name, kinetic model used to estimate half-life from concentration–time series, comment on half-life, SMILES of ^14^C-labeled spike compound, acidity (pH), cation exchange capacity (CEC), organic carbon (OC) content, biomass concentration at start and end of the experiment, temperature, type and value of water storage capacity, humidity, and soil texture.

### Curation of Data

Composite substances (i.e., salts) were manually checked, and the non-active parts of the molecule (e.g., Na^+^) were removed to retain only active substances. If several active substances were present in one compound entry, then the entry was removed. While the persistence of mixtures is out of the scope of this study, such data points could be used in the future to expand the approach to mixtures. Stereoisomers and duplicate compound entries were merged into single entries based on canonical SMILES without stereochemical information. Compounds were further annotated with InChI keys to facilitate identification. Half-life values were reported in days and converted to log units [log(DT_50_)]. For each substance, the mean, median, and standard deviation were calculated from log(DT_50_) (descriptive approach). The final, curated data set is available on GitHub along with the corresponding data curation workflow and as Tables S1 and S2.

### Bayesian Inference Model

Different experiments of the same substance *i* will report different half-lives DT_50_. To estimate the average log half-life *μ*_*i*_, we assume that the logarithm (base 10) of the reported half-lives of substance *i* can be described by a normal distribution
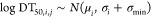
1where σ_min_ describes the minimal variability due to experimental conditions and σ_*i*_ describes the experimental noise. Unfortunately, the data are too sparse to explicitly derive the influence of different experimental conditions, particularly across the entirety of substances.^14^ We therefore estimated the minimal experimental variability from 27 reference substances for which the half-life distribution is well characterized by >20 data points. The mean standard deviation of the reference compounds was found to be 0.38 log(*d*), ranging from 0.2 to 0.7. Therefore, we set σ_min_ to 0.2 log(*d*) to avoid overconfident results.

We further consider that half-life data can be left- or right-censored. We assumed a global lower censoring threshold of 0.1 day and a global upper threshold of 1000 days. This choice is motivated by the OECD 307 guideline, which suggests concentration measurements at 1 day intervals in the beginning of the experiment, and an incubation time of ≤120 days.^8^ Reported half-life values that were extrapolated by more than one log unit, and therefore beyond the global censoring thresholds, are highly uncertain and considered here as left- or right-censored. In some cases, the half-life value is reported as beyond a specific threshold (e.g., >365 days). In this case, the indicated value is adopted as a local censoring threshold.

### Prior Definition and Justification

**Figure 1:**
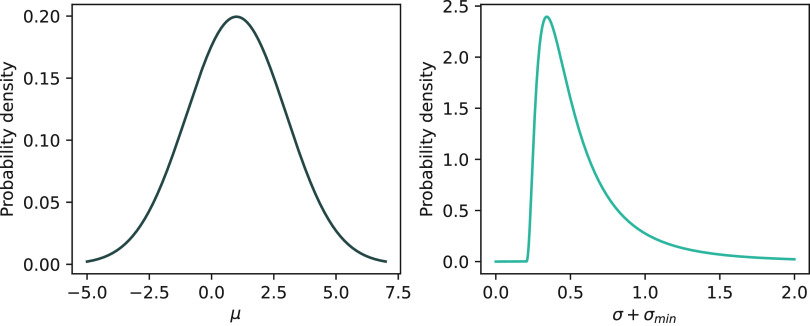
Prior distribution of μ and σ + σ_min_ for a range of plausible half-life values in log(*d*).

The prior distribution knowledge of the mean half-life (*μ*_*i*_) and the standard deviation of the experimental variability (*σ*_*i*_) are listed in Table 1. The chosen distributions (normal vs log-normal) reflect the observed distributions of the mean and standard deviation, respectively, of the half-life data. The parameters of the prior were chosen on the basis of the methodological constraints of the soil biodegradation studies and the reported half-life distributions of the reference substances (Table 1 and Figure 1). The prior for *μ*_*i*_ is centered in the measurable range [−1 to 3 log(*d*)], and a large standard deviation of 2 was chosen to cover the measurable range. The prior for *σ*_*i*_ is based on the reference data set previously used to determine σ_min_. For most compounds of the reference data set (59%), the standard deviation lies below the prior mean of 0.4, and hence, the prior is not too restrictive. Alternatively, one could choose a larger prior mean of the standard deviation of 0.6 as a worst-case scenario, which is larger than the standard deviation of >90% of the reference substances.

**Table 1:** Choices of Parameters for Prior Distribution

variable	distribution	mean [log(*d*)]	standard deviation [log(*d*)]
*μ*_*i*_	normal	1	2
σ_*i*_	log-normal	0.4	0.4

### Posterior Distribution and Sampling

The model based on eq 1 was implemented in Python, and for each substance in the data set, the posterior distributions of the average log half-life *μ*_*i*_ and the variability σ_*i*_ were inferred by Monte Carlo sampling. The posterior samples were summarized by mean and standard deviation. Samples of it were obtained with the Goodman & Weare’s Affine Invariant Markov chain Monte Carlo (MCMC) Ensemble sampler (https://github.com/dfm/emcee, version 3.1.3)^15^ using 10 walkers and 2000 iterations. A burn-in of 100 samples was discarded, and the remaining samples were used to calculate the mean half-life (*μ*_mean_), the uncertainty of the mean (*μ*_std_), and the experimental variability (*σ*_mean_).

## Results and Discussion

### Uncertainty Estimates and Inclusion of Censored Values for Soil Biodegradation Half-Lives

The EAWAG-SOIL data set contains 6309 half-life values for 893 compounds. Of these, 220 half-life values were beyond the global censoring thresholds (115 ≤ 0.1 day, and 105 ≥ 1000 days). Another 91 values were within the global censoring thresholds but specifically censored (78 on the left and 13 on the right) according to comments provided in enviPath. This means that upon removal of censored values, our data set would be reduced by 312 half-life values (5%) to 5998 and we would lose 21 substances (2%) for which only censored values are available. Bayesian inference allowed us to recover meaningful half-life estimations for these 21 substances.

**Figure 2:**
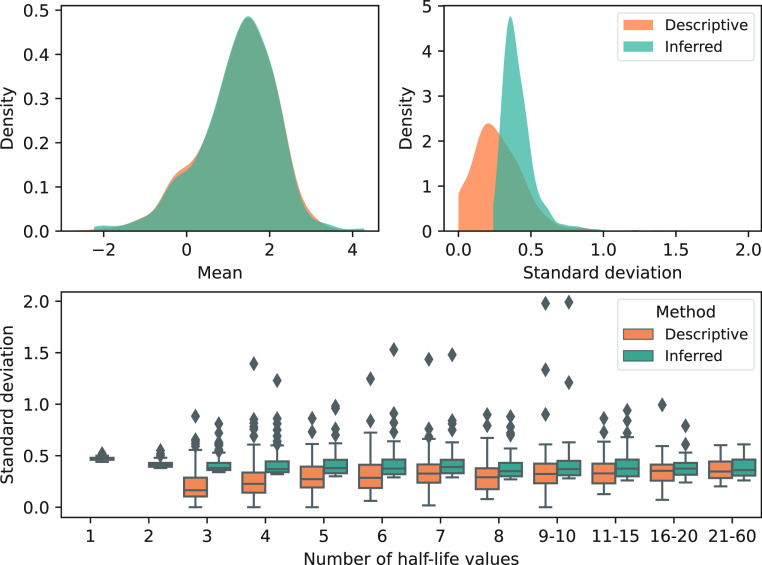
Distribution of soil biodegradation half-lives in log(*d*). Distribution of descriptive (orange) and inferred (blue) mean half-lives (top left). Distribution of descriptive (orange) and inferred (blue) standard deviations (top right). Distribution of descriptive and inferred standard deviations by the number of reported half-lives available for a given substance (bottom).

The average half-life *μ*_*i*_ of all substances is 1.23 log(*d*) for the descriptive approach and Bayesian inference (Figure 2). While the distributions of *μ*_*i*_ are similar for both approaches, the distributions of the *σ*_*i*_ differ, with a mean *σ*_*i*_ of 0.30 log(*d*) for the descriptive approach and a mean *σ*_*i*_ of 0.42 log(*d*) for the Bayesian inference approach. The higher average variability estimated by the Bayesian approach is in line with the chosen mean for the prior of *σ*_*i*_. However, the difference between the two approaches decreases with an increasing number of data points for a given substance, suggesting that both methods converge under a high-data regime (Figure 2). Finally, Bayesian inference provides uncertainty estimates for 83 substances with only one or two half-life values, for which the calculation of a standard deviation is not possible with a descriptive approach. This analysis shows that Bayesian inference can reliably estimate the mean and uncertainty of data points, but its outcome is sensitive to the prior assumptions, in particular if only a few data are available. Hence, careful prior choice and justification are crucial.

### Posterior Distribution for Different Example Cases

**Figure 3:**
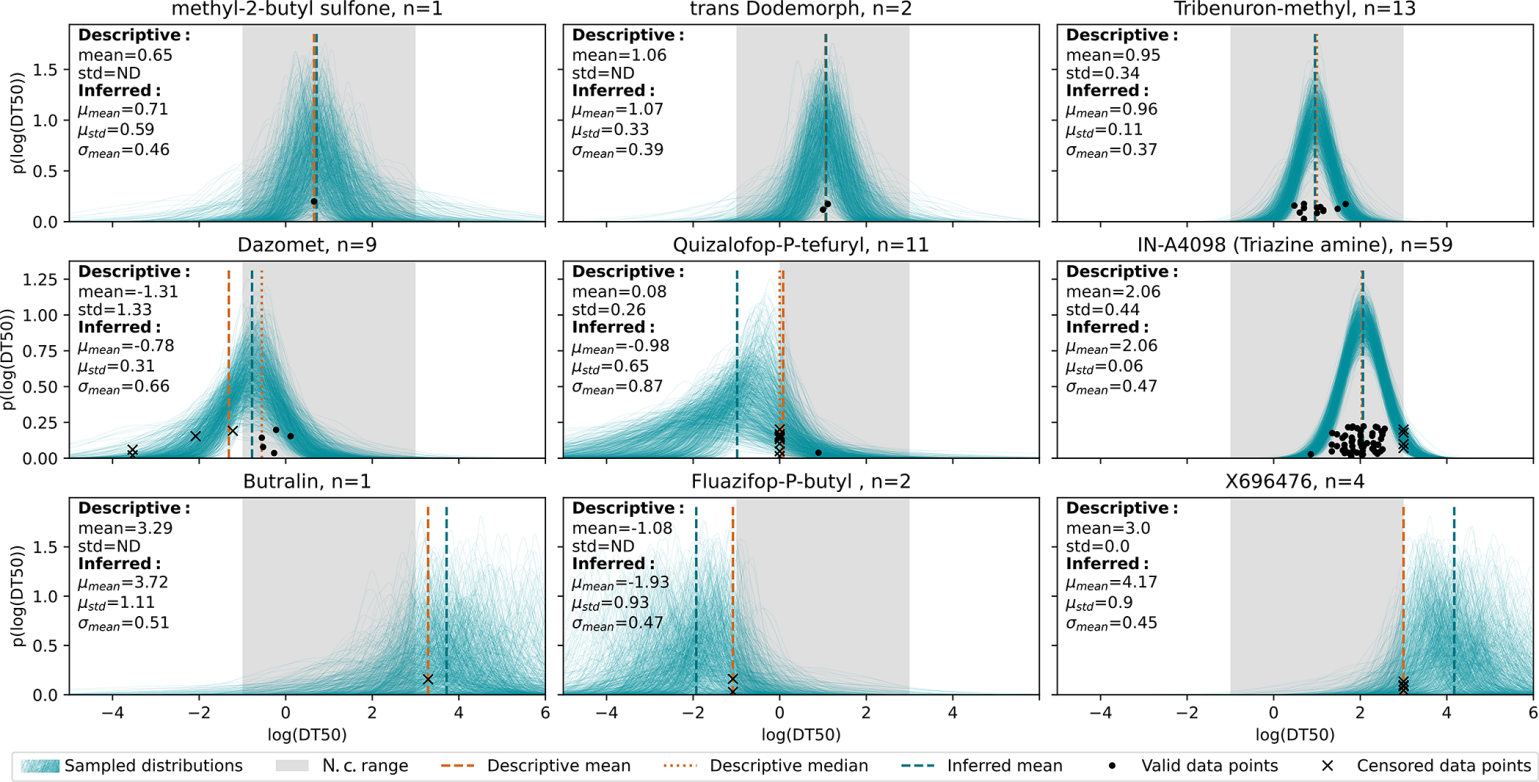
Different cases of data availability and how they affect the inferred distribution. Single reported half-lives [in log(*d*)] are shown as scattered data points along the *y*-axis. Dashed orange lines show the descriptive mean and median, and dashed blue lines show the inferred mean; the range of reasonable half-life values lying within the censoring thresholds is colored gray. To visualize the inferred distributions and their uncertainty, 1000 randomly chosen MCMC samples are visualized as normal distributions (thin blue lines). Abbreviations: std, standard deviation of reported half-lives; ND, not defined; N.c., not censored.

To illustrate the outcome of our approach, we present the results for nine substances representing different cases of data availability (Figure 3). In a low-data regime, Bayesian inference yields high uncertainty estimates of the mean (*μ*_std_). When many data points are available for a single substance, the probabilistic and the Bayesian inference distributions converge, and the uncertainty of the mean decreases. The variability estimates (*σ*_mean_) increase when data points show a higher spread and remain close to the prior otherwise. When data points lie beyond the censoring thresholds, so does the Bayesian-inferred mean with a decreasing uncertainty and variability when more censored data points are added.

It is important to note the difference between estimated mean experimental variability *σ*_mean_ and the uncertainty of estimated mean half-live *μ*_std_. The former describes the estimated variability of experimental data and is not necessarily reduced by the addition of more data points. On the contrary, the standard deviation of the MCMC samples *μ*_std_ can be interpreted as an indicator of the confidence in the inferred *σ*_mean_, and it always decreases with the addition of data points. For example, the compounds methyl-2-butyl sulfone and IN-A4098 (triazine amine) have a similar *σ*_mean_ of 0.47 log(*d*). However, the distribution of the first compound is inferred using only one data point, and therefore, the confidence in the inferred *μ*_*i*_ is low [*μ*_std_ = 0.56 log(*d*)]; for the second compound, *μ*_*i*_ is inferred on the basis of 59 data points and therefore the confidence is high [*μ*_std_ = 0.06 log(*d*)]. Hence, *μ*_std_ describes the uncertainty of the inferred mean and is particularly useful for evaluating the need for more experiments for a given compound.

### Applications in Half-Life Modeling and Regulation

The presented method using soil half-life data as an example can be readily adapted for application to biodegradation half-lives in activated sludge, water, or water/sediment systems, with an appropriate re-evaluation of prior assumptions. For example, Hofman-Claris and Claßen^16^ proposed a simplified determination of water degradation half-lives for highly persistent substances using unlabeled spike compounds, aiming to efficiently pinpoint persistent compounds with half-lives above the persistence threshold (DT_50_ > 40 days). This type of study is cheaper and faster than experiments with radiolabeled spike compounds, but it systematically results in censored values, therefore requiring appropriate statistical tools such as Bayesian inference for the correct interpretation of data.^10^

We suggest that the method might also be advantageous for analyzing data sets on toxicological, bioaccumulation, or mobility indicators or other types of environmental data with high variability and uncertainty. Data sets for training machine learning models can particularly benefit from reliable variability and uncertainty estimates. Uncertainty estimates could also impact regulation because they address the “threshold problem”. In regulatory hazard assessment, a substance is classified as persistent if its half-life value lies above a regulatory threshold.^17^ However, for the same substance, reported half-life values may lie above and below the threshold or a single measurement may lead to an erroneous persistence classification. These issues could be addressed by including a Bayesian uncertainty estimate to classify substances with an associated confidence metric. For example, a substance could be considered nonpersistent if 95% of experiments would indicate nonpersistence (usage of *σ*_mean_), or it could be considered nonpersistent if there is a 95% probability that its average half-life lies below the persistence threshold (usage of *μ*_std_). In this regulatory context, however, the definition of the prior distribution might be decisive, and further research and delibration among stakeholders would be needed to develop a methodology for defining defensible prior distributions.

Beyond the methodological demonstration of how Bayesian inference can be applied to derive distributions to deal with variable environmental end points transparently and reproducibly, we further provide here a curated data set of pesticide soil biodegradation half-lives compiled from regulatory reports, including descriptive as well as inferred half-lives. We also provide all necessary code to reproduce the extraction, curation, and analysis of data. We hope that this work will support future efforts to improve or validate half-life prediction models and to make statistically informed, robust regulatory decisions.

## Supporting Information Available

Original half-life values as extracted from the EAWAG-SOIL database, including experimental parameters (Table S1), and half-life values summarized per compound, including descriptive (mean, median, and standard deviation) and inferred half-life values (Table S2) (XLSX)

## Notes

The preprint of this paper has been submitted to *chemRxiv* (Hafner, J.; Fenner, K.; Scheidegger, A. Systematic Handling of Environmental Fate Data for Model Development - Illustrated for fhe Case of Biodegradation Half-Life Data. *chemRxiv* 2023. 10.26434/chemrxiv-2023-604k3).

## References

[ref1] EU, Regulation (EC) No 1907/2006 of the European Parliament and of the Council of 18 December 2006 concerning the Registration, Evaluation, Authorisation and Restriction of Chemicals (REACH), establishing a European Chemicals Agency, amending Directive 1999/45/EC and repealing Council Regulation (EEC) No 793/93 and Commission Regulation (EC) No 1488/94 as well as Council Directive 76/769/EEC and Commission Directives 91/155/EEC, 93/67/EEC, 93/105/EC and 2000/21/EC. Official Journal of the European Union 2006, 1–849.

[ref2] EU, Regulation (EC) No 1107/2009 of the European Parliament and of the Council of 21 October 2009 concerning the placing of plant protection products on the market and repealing Council Directives 79/117/EEC and 91/414/EEC. Official Journal of the European Union 2009, 1–50.

[ref3] EMA Revised Guideline on Environmental Impact Assessment for Veterinary Medicinal Products in Support of the VICH Guidelines GL6 and GL38. EMEA/CVMP/ERA/418282/2005-Rev.1; Committee for Medicinal Products for Veterinary Use (CVMP). European Medicines Agency (EMEA): London, 2009.

[ref4] EMEA Guideline on the Environmental Risk Assessment of Medicinal Products for Human Use; Committee for Medicinal Products for Human Use (CHMP). European Medicines Agency (EMEA): London, 2006.

[ref5] Commission Delegated Regulation (EU) 2023/707 of 19 December 2022 amending Regulation (EC) No 1272/2008 as regards hazard classes and criteria for the classification, labelling and packaging of substances and mixtures (Text with EEA relevance). 2022, Vol. 093.

[ref6] Arp H. P. H.; Hale S. E. Assessing the Persistence and Mobility of Organic Substances to Protect Freshwater Resources. ACS Environ. Au 2022, 2, 482–509. 10.1021/acsenvironau.2c00024. 36411866 PMC9673533

[ref7] Lorenz S.; Amsel A.-K.; Puhlmann N.; Reich M.; Olsson O.; Kümmerer K. Toward Application and Implementation of in Silico Tools and Workflows within Benign by Design Approaches. ACS Sustainable Chem. Eng. 2021, 9, 12461–12475. 10.1021/acssuschemeng.1c03070.

[ref8] OECD. Test No. 307: Aerobic and Anaerobic Transformation in Soil. 2002.

[ref9] Generic guidance for Estimating Persistence and Degradation Kinetics from Environmental Fate Studies on Pesticides in EU Registration.

[ref10] Helsel D. R. Statistics for Censored Environmental Data Using Minitab and R; John Wiley & Sons, 2011.

[ref11] Box G. E. P.; Tiao G. C. Bayesian Inference in Statistical Analysis; John Wiley & Sons, 2011.

[ref12] van de Schoot R.; Depaoli S.; King R.; Kramer B.; Märtens K.; Tadesse M. G.; Vannucci M.; Gelman A.; Veen D.; Willemsen J.; Yau C. Bayesian statistics and modelling. Nat. Rev. Methods Primers 2021, 1, 1 10.1038/s43586-020-00001-2.

[ref13] Latino D. A. R. S.; Wicker J.; Gütlein M.; Schmid E.; Kramer S.; Fenner K. Eawag-Soil in enviPath: a new resource for exploring regulatory pesticide soil biodegradation pathways and half-life data. Environmental Science: Processes & Impacts 2017, 19, 449–464. 10.1039/C6EM00697C. 28229138

[ref14] Wang Y.; Lai A.; Latino D.; Fenner K.; Helbling D. E. Evaluating the environmental parameters that determine aerobic biodegradation half-lives of pesticides in soil with a multivariable approach. Chemosphere 2018, 209, 430–438. 10.1016/j.chemosphere.2018.06.077. 29936116

[ref15] Foreman-Mackey D.; Hogg D. W.; Lang D.; Goodman J. emcee: The MCMC Hammer. PASP 2013, 125, 306 10.1086/670067.

[ref16] Hofman-Caris R.; Claßen D. Persistence of gabapentin, 1H-benzo-triazole, diglyme, DTPA, 1, 4-dioxane, melamine and urotropin in surface water: Testing of chemicals according to the OECD 309 guideline. KWR, 2020.

[ref17] REACH, 2017: Guidance on information requirements and chemical safety assessment, Chapter R.11: PBT/vPvB assessment, European chemicals agency (ECHA); COMMISSION DELEGATED REGULATION (EU) 2023/707 of 19 December 2022 amending Regulation (EC) No 1272/2008 as regards hazard classes and criteria for the classification, labelling and packaging of substances and mixtures. Official Journal of the European Communities 2023, 66, 7–39.

